# Substance P/Neurokinin 1 and Trigeminal System: A Possible Link to the Pathogenesis in Sudden Perinatal Deaths

**DOI:** 10.3389/fneur.2017.00082

**Published:** 2017-03-13

**Authors:** Riffat Mehboob

**Affiliations:** ^1^Biomedical Sciences, King Edward Medical University, Lahore, Pakistan

**Keywords:** sudden infant deaths, sudden perinatal death, substance P, neurokinin 1 receptor, trigeminal nerve

## Abstract

Sudden demise of a healthy fetus or a neonate is a very tragic episode in the life of parents. These deaths have been a mystery since ages but still remain unexplained. This review proposes the involvement of trigeminal nerve, neurotransmitter substance P (SP), and its receptor neurokinin 1 (NK-1R) in regulation of cardiorespiratory control in fetuses and newborns. Anomalies and immaturity of neuroregulatory systems such as trigeminal system in medulla oblongata of brainstem may provide a possible mechanism of sudden perinatal deaths. Vulnerable infants are born with respiratory center immaturity which in combination with any stressor such as cold, hypoxia, and smoking may lead to cessation of breathing and ventilatory response. SP/NK-1R may be involved in regulating the ventilatory control in neonates while it is decreased in fetal and adult life in humans, and any alterations from these may lead to irreversible sleep apnea and fatal breathing, ultimately sudden death. This review summarizes the studies performed to highlight the expression of SP or NK-1R in sudden perinatal deaths and proposes the involvement of trigeminal ganglion along with its nerve and SP/NK-1R expression alteration as one of the possible pathophysiological underlying mechanism. However, further studies are required to explore the role of SP, NK-1R, and trigeminal system in the pathogenesis of sudden infant deaths, sudden intrauterine deaths, stillbirths, and sudden deaths later in human life.

## Introduction

Sudden perinatal deaths comprise of stillbirths, fetal [sudden unexplained intrauterine deaths (SUID)], and infant death [less than 1 year of age, sudden infant deaths (SIDS)] that is spontaneous and mysterious. These cases remain unresolved even after complete autopsy, medical examination, and thorough investigation of death scene (1). Theories on the possible causes of SIDS are more than 100. It is widely accepted that SIDS is a combination of multifactors that occur during the period of increased vulnerability and may cause the fatal outcome in some infant (2). Bergman et al. also suggested that SIDS is not caused by “single characteristic that ordains an infant for death,” but depends on an “interaction of risk factors with variable probabilities” (3). Pathophysiology of SIDS remains unexplained (2).

Brainstem anomalies as a possible cause of sudden perinatal death is mostly accepted hypothesis as suggested by Filiano and Kinney (4) in their famous triple risk model. These neuropathologies lead to vulnerable fetus or newborn who becomes unable to respond to any kind of stressor and dies suddenly (5). All the factors whether maternal, infant, environmental, or genetic interfere with the cardiorespiratory control leading to final common pathway (death) (6). Mechanisms underlying SIDS appear to originate in fetal period of development resulting in neural damage and affect breathing or blood pressure during sleep later on (5). Neuromodulators like somatostatin, serotonins (7), substance P (SP), etc. regulate the breathing control activities (8–10). The focus of current review is to highlight the SP and its receptor NK-1R in neuronal respiratory control system during critical ontogenetic periods of human brain development. Our research group for the first time in 2011 (11) suggested the involvement of SP in the pathogenesis of SUID.

## Substance P

Substance P is the prototype and first discovered tachykinin. It is a neurotransmitter of the afferent sensory nervous system (12). It is a small peptide hormone consisting of 11 amino acids belonging to tachykinin family (TK) (13). It is the most abundant TK peptide and neurotransmitter in CNS of mammals (14). It has been implicated in various physiological and pathophysiological processes (15) and found in many central and peripheral neural pathways. SP is released from fifth cranial nerve, the trigeminal, which is part of trigeminal system that is explained below.

## Trigeminal System

**Figure 1:**
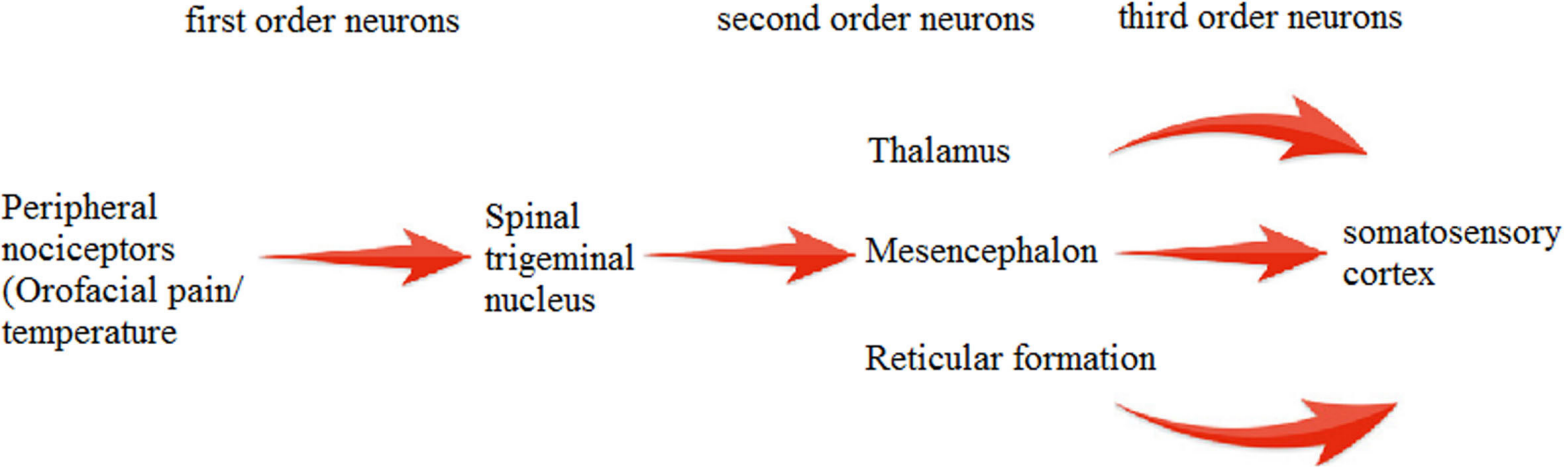
**Processing of nociceptive stimulus within brain**.

Trigeminal system is highly established and well-studied system in mammals and birds (16). SP immunoreactivity (SP-IR) has been observed in trigeminal (17) and dorsal root ganglia (DRG) (18). Main feature of this system is the presence of two distinct primary afferent neuronal groups: trigeminal ganglion (TG) and mesencephalic trigeminal nucleus (MTN). Cell bodies of these primary afferent neurons are present in TG (19), and few lie in MTN. MTN is involved mainly in proprioception (mainly orofacial musculature) (20). TG dorsomedial part is involved in nociception, thermoreception, and proprioception while its ventrolateral part is involved in mechanoreception (21). Signals from the trigeminal system are transmitted by second order neurons in brainstem to different regions of CNS pain centers (22). The processes in the middle of ganglion end up on various groups of second order neurons, which convey their signals to the somatosensory cortex *via* thalamus (23) (Figure 1).

## Trigeminal Ganglion

Trigeminal ganglion is accumulation of pseudounipolar neurons (24) and consists of neurons and their fibers. TG is a cranial analog of DRG in PNS (21). Activation of TG nerves plays a central role in most forms of orofacial pain (25). Many neurotransmitters and their receptors are localized in different subpopulations of TG neurons (21). TG neurons supply innervations mostly to the mechanoreceptors, thermoreceptors, and nociceptors in orofacial region (16). Glial cells also known as satellite cells (26) completely enclose the neuronal somata of TG neurons, and thus they have no synaptic contacts (27).

Based on their appearance, ganglion cells are classified as follows: large light (A) and small dark (B) cells (28). Large light A cells produce thick myelinated fibers while the thin C fibers (both myelinated and unmyelinated) are originated from small dark B cells (29). Two primary afferent neuron subpopulations are noticed in TG: small- and medium-sized neurons with small somata, including glutamate, somatostatin, SP, neurokinin A, CGRP, cholecystokinin, vasoactive intestinal peptide, and galanin, and larger sized neurons that are relatively less and include neuropeptide Y and peptide 19. Presence of SP in small diameter primary afferent fibers and in nociceptive centers of brain gives us an idea of its nociceptive role (30).

## Trigeminal Nerve (TrN)

**Figure 2:**
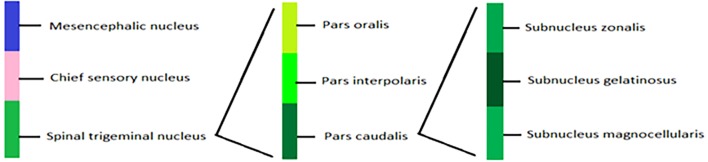
**Graphical representation of trigeminal nerve nucleus**.

Trigeminal ganglion provides somatosensory innervations of face and oral cavity through TrN (31) (Figure 2). It has three branches: V1 and V2 are purely sensory while V3 has both sensory and motor functions. V1 innervates forehead, upper eyelid, cornea, conjunctiva, mucosa of frontal ethmoid and sphenoid sinuses, and dorsum of nose. V2 innervates upper lip, lateral portions of nose, parts of oral cavity, mucosa of nasal cavity, maxillary sinus, upper jaw, and roof of mouth and upper dental arch while V3 innervates lower lip, chin, cheek, lower teeth, gingival, mucosa of lower jaw, floor of mouth, and anterior two-thirds of tongue (25).

## Neurokinin Receptors

Tachykinin receptors also known as neurokinins (NK) are G protein-coupled receptors, localized in the nucleus of the solitary tract, which is known to be involved in the rhythmic control (32). There are three NK receptors (1R-3R). Functional activities of SP are initiated after binding to the neurokinin 1 (NK-1R), which is a transmembrane protein (33). Upon binding, a chain of signaling events is activated by the internalization of SP–NK-1R complex (34), which stimulates the second messenger phospholipase C resulting in the production of inositol 1,4,5-trisphosphate (IP3) and diacylglycerol (DAG) (35). Calcium is released intracellularly as a result of stimulation of endoplasmic reticulum by IP3, and protein kinase C is activated by DAG (36).

These neurokinin receptors are present within the cardiorespiratory regulatory control centers and in the phrenic nucleus, which controls the diaphragm and mediates the respiratory responses to SP. Prototype TK, SP, was reported to stimulate the respiratory rhythm in wild-type mice, in the *in vitro* brainstem–spinal cord preparation but not in the NK-1R knockout (NK-1R−/−) mice (37). The study of *in vitro* brainstem preparations revealed that NK-Rs have a vital role in the regulation of respiratory control and lung burst activity during the development of bullfrog from tadpole to adult stage (38). Role of SP has been implicated in the development of plasticity of respiratory system and the regulation of respiratory rhythm. A functional SP-ergic system is necessary for the generation of sufficient ventilatory responses to hypoxia in newborn mice and during early maturation (39). Under increasing hypoxia, SP manifests as natural anti-hypoxant and is not only involved in nociception mechanisms but also in brain adaptation to oxygen deficiency (40). SP-ergic system was found to be more active in regulating the respiratory responses during the early postnatal period in neonatal rat brainstem–spinal cord preparation (41) and medullary slice preparations of newborn mice (42). But surprisingly, SP was not found to control ventilatory rhythm generation in fetal rats, and it was hypothesized that, may be, SP does not modulate the generation of respiratory responses before birth and affects the phrenic motoneurons only after birth (43).

## Role of SP/NK-1R in Regulation of Respiratory Rhythm

Neuromodulator SP causes dilation of vessels, contraction of smooth muscles in the respiratory system, increased action potential in neurons, an increase in vascular flow, and production of saliva (44). In humans, NK-1R is involved in causing bronchoconstriction (45). NK-1Rs mediate increases in secretion of mucous glands in human trachea (46). SP-IR was observed to be potentiated in a study conducted on the bronchoalveolar lavage fluid (47) and sputum samples (48) from asthmatic patients. NK-1R mRNA expression was also higher in asthmatic lung tissue when compared to non-asthmatic (49). Elevated levels of SP and PPT-A mRNA were observed in the nodose ganglia of ovalbumin-sensitized guinea pigs (50) with increased neurogenic inflammation and bronchoconstriction produced by NK-1R (51). It suggests that SP/NK-1R and neurogenic immunoreactivity are critical for the progression of airway hyperresponsiveness (AHR) (52), and NK-1R antagonists attenuated the AHR and plasma extravasation in animal models *in vivo* (51). The underlying mechanism in causing AHR may be the airway inflammation and interaction of SP and CGRP (calcitonin gene-related peptide) (53). Mice deficient in NK-1Rs show reduced IgG-mediated lung injury and neutrophil infiltration as compared to the control group (54). SP is present in bronchopulmonary C fibers (PCFs) and defend the lungs against injury from inhaled agents by a CNS reflex consisting of apnea, cough, bronchoconstriction, hypotension, bradycardia (55), secretion from seromucous glands, release of mediators (including prostaglandins and NO) from the airway epithelium (56), and bronchorelaxation (57). SP synthesis in vagal airway C fibers may be enhanced in pathological conditions such as allergic asthma and chronic bronchitis and may be responsible for some of the associated respiratory symptoms stated above (55). SP produces bronchoconstriction and lung resistance in cynomolgus monkeys (58) and sheep (59) *via* NK-1 receptors, and this effect is more pronounced when they are given by the intravenous route.

## Evidence of Involvement of Spinal Trigeminal Nucleus in Respiratory Rhythmic Control

Few earlier studies with small sample size have been performed in relation to SP expression in brainstems of SIDS (60–63). Our group (11) defined localization, morphology, and functional aspects of TrN in different developmental stages of human brain development for the first time in cases of sudden perinatal death victims. This study revealed that fetuses in control group had no well-defined TrN as there was either weak or no SP expression, while in medulla oblongata sections from control infants, a well-defined TrN with recognizable tract was observed. While SP expression was depleted in SIDS victim brainstems showing TrN hypoplasia and enhanced in SUID victims. Density of SP varied from very low in fetuses to very high in infants, which points out to the functional requirement of TrN in postnatal period of human life. TrN development is enhanced in late developmental stages of brain, which was identified by enhanced SP expression. Recently, trigeminocardiac reflex has been suggested as an underlying mechanism for the pathogenesis of SIDS (7).

I have also reported in a previous computational study that SP/NK-1R has small protein interaction network, its gene is singleton, providing a possibility that it may be involved in some extremely crucial activities of human developmental phases, and any perturbation in terms of mutations in the gene can lead to lethal outcomes including sudden death (64). Similarly, in another study, I along with my colleagues observed higher expression of SP in oral cancer patients from Pakistani population. This expression was directly proportional to the grade of cancer and poor prognosis. This study also confirms the involvement of SP in nociceptive stimuli in orofacial region (65). In our previous study (11), we detected SP expression in medulla oblongata and spinal cord from early stages of human brainstem development, exclusively in the spinal TrN. This phenomenon exhibits that SP is highly specific and localized in human brain. Very few studies focus on the TrN in humans (66, 67). As spinal trigeminal nucleus and its tract have no definite boundaries, it is difficult to identify it. Nevertheless, it can be recognized by labeling it with SP, which is its main neurotransmitter (11). In experimental murine studies, TrN was observed to be involved in rhythmic autonomic behaviors including breathing (68, 69). These studies also suggested an essential role of TrN in controlling respiratory rhythm, and its activity was observed to be altered in respiratory disorders (70, 71).

**Figure 3:**
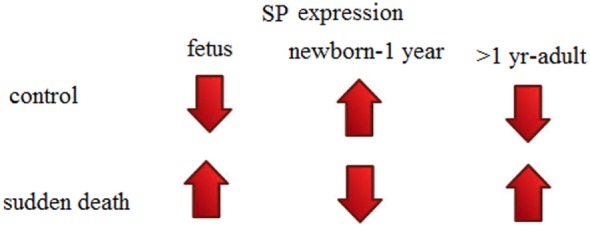
**Substance P (SP) expression theory (in human brainstems) to explain sudden perinatal deaths**.

Observations from a recent study by Hayashi and Sakuma (72) are also concordant with our observations and findings that SP expression was elevated in seven out of eight victims of sudden unexplained deaths. A previous study also suggested that SP containing cells in human TG is variable in different age groups; 24% in neonates while 17% in adults (73). Study of Hayashi and Sakuma (72) shows expression of SP in brainstem of children and adults as compared to our study, which reported SP expression in SIDS and SUID. If we summarize the findings of both studies and compare them with our study also, we reach to conclusion that SP expression in brainstems is negligible in fetus, enhanced in neonates while decreased in children and adults in controls. While if *vice versa* it leads to sudden deaths (Figure 3).

## Conclusion

Studies suggest an important role of SP/NK-1R and trigeminal system during the critical neurodevelopmental periods in which brain is preparing to deal with vital autonomic functions such as breathing, required for the start of new life after birth. Trigeminal nucleus hypoplasia and variations in expression of SP in sudden perinatal death victims as compared to controls provide a possibility of revealing one of the underlying mechanisms in its pathophysiology.

## Author Contributions

RM has designed, planned, written, and contributed to this review manuscript.

## Conflict of Interest Statement

The author declares that the research was conducted in the absence of any commercial or financial relationships that could be construed as a potential conflict of interest.
